# Weekly Fluctuations of Plasma Amyloid‐Beta in Alzheimer's Disease: Implications for Biomarker Reliability

**DOI:** 10.1002/alz70861_108714

**Published:** 2025-12-23

**Authors:** Andrew Pickering, Carolyn Pickering, Andy Delgado, Kanisa Davidson, Danitra Parker, JaLeesa Bryant

**Affiliations:** ^1^ University of Texas Health Houston, Houston, TX USA; ^2^ University of Texas Health Science Center at Houston, Houston, TX USA; ^3^ the university of texas health science center at houston, Houston, TX USA; ^4^ The University of Texas Health Science Center at Houston, houston, TX USA; ^5^ The University of Texas Health Science Center at Houston, Houston, TX USA

## Abstract

**Background:**

Alzheimer's Disease (AD), the most prevalent age‐related dementia, is characterized by the accumulation of Aβ and hyperphosphorylated tau. Accurate monitoring of AD progression is crucial for patient management and the evaluation of clinical trials, but often involves invasive and expensive methods like cerebrospinal fluid analysis and PET scans. Consequently, non‐invasive serum biomarkers such as Aβ42/Aβ40 have gained popularity in clinical trials and clinical research. This study investigates the variability in plasma Aβ levels on a micro‐longitudinal scale, challenging the assumption of their stability and exploring the implications of their use in clinical trial monitoring.

**Method:**

Aβ42 and Aβ40 peptides were quantified in plasma by immunoassays in a cohort of 73 AD patients with AD8 scores ≥3. Our cohort was‐62% Black/African American and 38%‐White/Caucasian, 66%‐Female and 34%‐male. Samples were collected weekly over a five‐week micro‐longitudinal study. We examined week‐to‐week in Aβ peptide levels within‐participant as well as between‐patient variation. Impacts both of sex and race were examined as covariates.

**Result:**

Significant week‐to‐week fluctuations were observed in the levels of Aβ40, Aβ42, and Aβ42/ Aβ40. We observed Aβ42/40 ratio to shift by more than 50% in 60% of participants. Further participants had an average of 20‐50% fluctuations in readings on a week to week basis. This week‐to‐week fluctuation appeared to be driven primarily by sudden episodic shifts in Aβ content with only a few individuals showing sustained shifts. We report no significant different in fluctuations in Aβ content by sex or race of participants. However we do did find blood amyloid content (Aβ42, Aβ40 and Aβ42/Aβ40) to be elevated in male participants. Finally, we show some participants to present higher levels of amyloid flux than others at least on a micro‐longitudinal scale. These findings highlight the dynamic nature of plasma Aβ levels, contrasting with the expected stability over such a short period.

**Conclusion:**

The substantial weekly variability in plasma Aβ levels suggests that relying solely on these biomarkers for AD monitoring or outcome assessment could be misleading without considering their temporal fluctuations. This study emphasizes the need for frequent sampling and cautious interpretation when using plasma Aβ levels as an outcome measure.

